# The role of preschoolers’ home literacy environment and emergent literacy skills on later reading and writing skills in primary school: A mediational model

**DOI:** 10.3389/fpsyg.2023.1113822

**Published:** 2023-03-01

**Authors:** Lucia Bigozzi, Giulia Vettori, Oriana Incognito

**Affiliations:** Department of Education, Languages, Interculture, Literatures and Psychology, University of Florence, Florence, Italy

**Keywords:** home literacy environment and practices, notational skills, phonological skills, reading, writing, longitudinal research

## Abstract

The importance of the quality of home literacy environment and practices (HLE&P) in the earliest years on children’s reading and writing development is recognized in the literature. However, whether and to what extent this relationship between preschoolers’ HLE&P on their later reading and writing skills in primary school is mediated by emergent literacy competence remains to be clarified. It may be that preschool constitutes a significant opportunity for children to develop notational awareness and phonological awareness which are emergent literacy skills that are fundamental for later reading and writing skills. Children who experience literacy-poor HLE&P with fewer opportunities to practice more complex language skills and diverse vocabulary might develop adequate reading and writing skills when their emergent literacy skills in preschool are high (notational and phonological awareness). This longitudinal study aimed to investigate the mediational role of preschoolers’ emergent literacy skills in preschool (notational and phonological awareness) in the relationship between HLE&P and reading and writing skills shown by the same children in primary school using a large-scale dataset. A total of 115 children (mean-age at last year of preschool = 4.88 ± 0.36) took part in the research. In preschool, children performed emergent literacy tasks and their parents completed a home literacy questionnaire. Later, in primary school, children completed standardized assessments of spelling (orthographic accuracy and fluency in a dictation task) and reading decoding (accuracy and speed in a text reading task) skills. The results of mediational analyses showed that notational awareness totally mediates the relationship between HLE&P and reading speed (𝛽= − 0.17, *p* < 0.05) and writing accuracy (𝛽=0.10, *p* < 0.05), but not for reading accuracy in primary school. The mediational model with phonological awareness as mediator was not significant. The results are discussed in the light of the effect of preschool in contributing to filling children’s home literacy gaps and disadvantages. In preschool, emergent literacy programs are essential to counterbalance the needs of preschoolers to develop adequate reading and writing skills when the family cannot provide enriched HLE&P from the early years of life.

## 1. Introduction

The importance of the quality of home literacy environment and practices (HLE&P) in the earliest years on the development of emergent literacy skills (Incognito and Pinto, 2021) and on children’s reading and writing development (Kim, 2009) is recognized in the literature. Children in preschool develop important emergent literacy skills such as notational awareness and phonological awareness which are fundamental for later reading and writing skills (Pinto et al., 2009, 2016; Bigozzi et al., 2016a,b). Mol and Bus (2011) conducted a meta-analysis and found that shared book reading with preschoolers supported young children’s language, reading, and spelling growth over time. However, whether and to what extent the relationship between preschoolers’ HLE&P on their later reading and writing skills in primary school is mediated by emergent literacy skills remains to be clarified. As highlighted by meta-analysis in this field of research (Zhang et al., 2021) most studies used cross-sectional designs limiting our knowledge of long-term associations between HLE&P in the emergent literacy period and later formalized literacy outcomes. Moreover, the interconnections between children’s home literacy factors and different environmental settings, such as preschool, require further investigations to understand the impact of home literacy on later reading and writing development. Therefore, we developed a longitudinal study aimed at examining the mediational role of preschoolers’ emergent literacy skills in preschool in the relationship between HLE&P and reading and writing skills shown by the same children in primary school using a large-scale dataset.

### 1.1. Home literacy and emergent literacy skills in preschool

In this study, we refer to a model of reading and writing development in primary school closely linked to the previous important literacy experiences that young children have at home (Kim, 2009) and at preschool (Pinto et al., 2012). As documented in previous studies (Sénéchal and LeFevre, 2002), in fact, the predictors detected that the acquisition of reading and writing skills is best conceived as a *continuum* that begins in the preschool period and continues along the formalized literacy period. According to Bronfenbrenner’s ecological theory (1979), it is possible to trace young children’s reading and writing development by referring to the microsystem of the family where important informal home experiences related to literacy occur. Different theoretical frameworks have emerged in understanding the construct of *home literacy*. Comprehensively, models proposed in the literature (e.g., Sénéchal and LeFevre, 2002; Manolitsis et al., 2011) show that both code-related practices and meaning-related practices are important to fully conceive home literacy of children who are preschoolers. Code-related practices see a direct involvement of 4-to-6 years old children with print, such as invented spelling activities, meanwhile meaning-related activities focus on sharing and constructing meanings, such as 4-to-6 years old children’s telling stories. Thus, home literacy is an articulated construct that includes practices that are autonomously activated by the child (Sénéchal and LeFevre, 2002), as well as practices guided by the parent, some of which incidentally promote literacy (e.g., joint reading of stories), while others are intentionally intended to promote literacy (e.g., the parent teaching the child the alphabet) (Sénéchal et al., 1996, 1998). Different families may vary in their HLE&P, for example in terms of literacy materials and stimuli used by the family or in terms of literacy practices, for example the participation of children in a joint book reading session with their parents (Niklas and Schneider, 2015) or telling and inventing stories, all activities connected to a child’s cognitive, creative and lexical development (Noble et al., 2019). Most of the studies framed on the model of Home Literacy focus on a quite limited developmental span, usually ages 4–6. Moreover, there is the need to study home literacy in connection with preschool when young children have the opportunity to develop important emergent literacy skills for later reading and writing development, such as notational awareness and phonological awareness. Phonological awareness denotes 4-to-5 years old children’s ability to identify units of sounds in words and manipulate patterns of sound, such as in the preschool activity of developing rhyme and identifying similar sounds in words. Phonological awareness is a key competence across alphabetic orthographies, but it plays a stronger role in predicting literacy outcomes in opaque orthographies where there is an irregular correspondence between sound-sign (Georgiou et al., 2012; Moll et al., 2014) in comparison to regular orthographies. Strictly linked to phonological awareness, notational awareness denotes 4-to-5 years old children’s ability to translate sounds into appropriate written signs by using a phoneme-grapheme correspondence, such as the preschool activity of invented spelling. Prior studies have recognized the predictive role of phonological awareness and notational awareness for later reading and writing acquisitions, even if their predictive weight changes in relation to the specific characteristics of orthographies. In the transparent Italian language, the emergent literacy model validated by Pinto et al. (2009) was composed by preschoolers’ notational awareness, phonological awareness, and textual awareness. Subsequent longitudinal research results based on this model (Bigozzi et al., 2016a) showed the predictive value of the three key emergent literacy skills on later reading and writing acquisitions. These results confirmed the predictive role of 4-to-5 years old children’s phonological awareness on later literacy outcomes, even if 4-to-5 years old children’s notational awareness played a stronger predictive role on later reading and writing acquisitions in Grade 1 and 2 (Pinto et al., 2017) in comparison to phonological awareness, while textual competence uniquely predicted later text writing skills (Pinto et al., 2016).

It is important to investigate the interaction between preschoolers’ home literacy and emergent literacy skills in predicting primary school children’s reading and writing skills in the Italian language which has regular and transparent orthography with an almost biunivocal correspondence between grapheme and phoneme. Each of the five vowels has only one orthographic translation in Italian, regardless of the context in which they are reported. Consonants have only one orthographic translation with a few exceptions (e.g., stop consonants and affricates: /k/and/g/; /tʃ/and/dƷ/). Beyond a few cases in which the orthographic rendition of the word is phonologically unpredictable (e.g., the voiceless velar/k/followed by the vowel/u/is rendered in/kwadro/ [picture] as “quadro”). Preschoolers with high levels of notational awareness show the ability to master the reciprocal sound-sign correspondence at the basis of reading and writing words, the availability in memory of the orthographic representation of the letters of a word and ability to transfer all this knowledge to a sheet of paper (Sénéchal et al., 2001; Pinto et al., 2009) with positive repercussions on their later reading and writing acquisitions in primary school years (Ouellette and Sénéchal, 2017; Albuquerque and Alves Martins, 2022). Thus, it may be expected that preschoolers’ notational skills play a significant role in predicting reading and writing in interaction with home literacy. The predictive role of home literacy and emergent literacy skills in preschool must be tested in their simultaneous interaction, rather than in isolation, and across languages that differ in orthographic depth to confirm whether it is a language specific pattern or it is transversal across languages (Georgiou et al., 2012).

### 1.2. Reading and writing: The role of home literacy and emergent literacy skills

The close relationships between preschoolers’ HLE&P on emergent literacy skills and later reading and writing have been identified in previous studies.

Previous studies have shown a direct relationship between home literacy and emergent literacy skills in preschool. For example, based on conceptual models in the literature (Fritjers et al., 2000; Rodriguez and Tamis-LeMonda, 2011), Kim et al. (2015) used a composite measure of home literacy in toddlerhood (e.g., 4.5 years old) and found that it predicted both vocabulary and decoding skills measured at preschool age. One longitudinal study (Sénéchal and LeFevre, 2014) from preschool to the beginning of Grade 1 showed links between home literacy environment and reading in English.

Another longitudinal study conducted in the transparent Italian language system (Incognito and Pinto, 2021) showed links between home literacy assessed as a composite score in the last year of preschool and emergent literacy skills assessed at the beginning and end of the preschool year. Indeed, preschoolers with high levels in home literacy practices were more likely to show high oral narrative skills and notational awareness at the beginning of the last year of preschool, while this relationship was less evident for phonological awareness. A link between home literacy and preschoolers’ emergent literacy skills has also been found in Korean children of four and 5 years (Kim, 2009) since frequent reading at home was positively associated with children’s emergent literacy skills, as well as conventional literacy skills.

Research with preschoolers has also revealed connections between emergent literacy skills with later reading and writing acquisitions in the formalized literacy period in different language systems (e.g., Silinskas et al., 2020; Zhang et al., 2020). In consistent orthographies, like Italian, which have a biunivocal relationship between phoneme and grapheme, preschoolers who master the correspondence between sound-sign, named notational awareness, are more likely to succeed in later reading and writing tasks at school (Pinto et al., 2012). Also, preschoolers’ ability to identify and manipulate phonological segments in spoken words, named phonological awareness, have consistently been found to be closely associated with children’s reading development with different predictive weights depending on the characteristics of orthographies in transparent languages (Landerl et al., 2019). It is important to adopt a cross-linguistic perspective on the study of home literacy impact on early literacy development (Inoue et al., 2020). Taken together, a body of research has provided evidence of relationships between HLE&P with preschoolers’ emergent literacy skills and primary school children’s reading and writing skills.

These results suggest the importance of a future study in order to provide an accurate picture of the relationships between preschoolers’ HLE&P, emergent literacy skills, with later reading and writing skills using mediational models. Indeed, it would be desirable to promote a better understanding of the transition from emergent to formalized literacy period by considering the preschool period as a key period for reading and writing acquisitions. The longitudinal study by Inoue et al. (2018) showed the relationships between home literacy environment and emergent literacy skills in kindergarten, and different reading outcomes in primary school years in Canadian children learning to read English. Although the literature informs us about the relationships between home literacy and emergent literacy skills or between home literacy and later reading and writing development, there is a lack of knowledge about the mediational role of preschoolers’ emergent literacy skills in the relationship between HLE&P and later reading and writing skills in transparent orthographies like Italian.

## 2. This study

A longitudinal study was developed to investigate the pattern of relationships between HLE&P with emergent literacy skills (i.e., notational awareness and phonological awareness) measured in preschool, and later reading and writing skills measured in the same children in the first grade of primary school. We intended to explore this pattern of relationships using a longitudinal mediational model, rather than by the more commonly used direct model of analysis widely spread in the literature. The longitudinal research design allowed us to connect preschool, as a key period for reading and writing acquisitions, with the formalized school period that in the Italian educational context starts at 6 years and corresponds to the formalized teaching of reading and writing. Following models of emergent literacy, we assumed as mediators preschoolers’ notational awareness and phonological awareness, chosen for their significant predictive contribution to reading and writing found in previous studies conducted in the Italian transparent language system (Pinto et al., 2009; Bigozzi et al., 2016b).

We expected HLE&P to positively contribute to the development of emergent literacy skills in preschoolers and later reading and writing skills in primary school. More specifically, we expected that this relationship would take the form of a mediational model where preschoolers’ emergent literacy skills exert a significant mediational role between HLE&P measured in preschool and later reading and writing skills measured in primary school.

## 3. Methods

### 3.1. Participants

A total of 115 children (mean-age at the last year of preschool = 4.88 ± 0.36; male = 57% and female = 43%) attending public all-day preschools in Italy were followed longitudinally till the first year of primary school. *In a first step*, the children were tested when attending their last year of preschool. The Italian preschool is a 3-year program that involves children from 3 to 6 years with a curriculum that follows national guidelines established by the Ministry of Education. Italian preschool programs do not provide formal instruction in reading and writing, rather they include pre-reading and pre-writing activities with a multimodal modality (e.g., drawing, invented spelling, songs) with the aim of facilitating children’s knowledge of letters, letter-sound and letter-sign correspondences. Approximately 96% of Italian preschools are public and are attended by approximately 98% of children with a school week of about 40 h. The transition from preschool to primary school usually occurs in the same school district making it quite easy to follow children longitudinally over time. *In a second step*, the same children were tested 1 year later when attending their first grade in primary school when formal teaching of reading and writing occurs. The Italian primary school is a 5-year program that involves children from 6 to 10 years with a curriculum that follows national guidelines established by the Ministry of Education. In the Italian schooling system, the formal instruction of reading and writing starts at the beginning of the first year of primary school with the expectation that children can reach an adequate level of coding and decoding accuracy at the end of the year (Pinto et al., 2015).

Participants were recruited from public schools located in two small towns in central Italy. They came from families with medium socioeconomic backgrounds, in line with the 2022 budget estimates (ISTAT,^1^ 2022).

Children with any known special educational needs or impairments/disorders were excluded from analyses to avoid any additional difficulties that could potentially affect their performance.

School authorities, parents, and children gave consent to participate in the study.

### 3.2. Measures

#### 3.2.1. First step of assessment in preschool—Home literacy environment and practices

To investigate preschool children’s home literacy and practices, we used a questionnaire (see Incognito and Pinto, 2021) that required parents to determine the extent of the reading material that children between three and 5 years old had access to at home; parents’ habits with regard to reading with and to the child; parent’s opinions on how they approached the child’s development of oral language, writing and reading; parents’ observations on the child’s behavior with regard to written language; and how parents answered any questions on the subject. The questionnaire collected data on activities consistent with existing models, including activities in which both father and mother are involved. Examples of items were “When does your child receive books and/or periodicals?” or “Do you read books and/or periodicals to your child?” or “When faced with your child’s curiosity about written things, how do you deal with it? (e.g., do you tell him/her what is written in it; take cues to teach him/her the first rules of writing; etc.).” Responses were required on a 3-point scale noting the frequency of the behavior enacted. The range of responses was from 0 to 3 points. The questionnaire consisted of 15 items. Both parents separately completed the questionnaire. A composite score was generated by averaging the mother’s and father’s scores. Based on home literacy indices, the children were distributed as follows: 46% with low levels of home literacy and 54% with high levels of home literacy. In the data used in this study, Cronbach’s alpha coefficient for the composite measure of the HLE&P was 0.70.

#### 3.2.2. First step of assessment in preschool—Notational awareness

Notational awareness was evaluated with an invented spelling task (Bigozzi et al., 2016a). Children were asked to draw, write, and read aloud what they had written by following it with their finger. The task was administered individually, and each child was equipped with a pencil and a white A4 sheet of paper to perform the test, which consisted of seven items. The first item is familiarization in which the child is asked to write his/her name as he/she knows how and read it by following it with his/her finger.

The other six items encode three components of notational awareness, which shows whether the child is aware of the diversity between iconic representation and written sign, numerical variation, and variation in phonemic units.

Specifically, two items measured the conceptual knowledge of orthographic notation that shows whether children are aware that there is a specific and exclusive sign system, other than the iconic sign, for writing the sound stream. An example item was, “Can you draw an apple? Can you try to write *mela* (apple)? Can you try to read what you wrote by following it with your finger?”

Another two items measured conceptual knowledge of the orthographic variation of sound quantity showing whether the child is aware that the number of spoken word sounds and the number of written sounds. An example item was, “Can you try to write the shortest word and the longest word you know? Can you try to read what you wrote by following it with your finger?”

Finally, two items measured conceptual knowledge of the orthographic variation of phonemic units. This coding scheme shows whether children are aware that similar sounds require the affixation of similar signs and different sounds of different signs. An example item was, “Can you try to write *gatto* (cat) and *gatti* (cats)? Can you try to read what you wrote by following it with your finger?”

Each item was coded, and a mean score was calculated. Participants’ scores ranged from a minimum of 0 to a maximum of 3. The agreement index of independent judges was between 90 and 99%. In the data used in this study, Cronbach’s alpha coefficient for the composite measure of the notational awareness context was 0.85.

#### 3.2.3. First step of assessment in preschool—Phonological awareness

To assess phonological awareness, a task involving the identification and production of sound patterns was administered (Dowker and Pinto, 1993; Pinto et al., 2009). Children were exposed to two verbal stimuli, one containing rhymes, and the other containing a series of alliterating words. Children were asked to listen to a short poem and invent a similar poem, with the stimuli acting as examples.

According to the Dowker and Pinto task, examples of Italian stimulus poems included: (1) Rhyming: II gatto Martino/ Uscendo il mattino/ Scendendo le scale/ Si fece del male. (“The cat Martino/Getting up in the morning/and going downstairs/Hurt himself.”); (2) Alliterating: Per una strada/Stretta e storta/Una strana cavalla/Trotta stanca. (“Along a street/Narrow and crooked/a strange horse/trotted wearily.”)

Three scores were derived for rhythm (children’s ability to reproduce the prosody), rhyme (children’s ability to detect the rhymes within the stimulus), and alliteration (children’s ability to detect alliterations within the stimulus); specifically, the rhythm of a poem is given by the way the lines are structured. By rhyme we mean when two or more words share sounds at the end of the word itself, usually based on a corresponding vowel and the sound that follows it. Whereas, alliteration is a repetition of letter sounds within words. A score of 0 indicated no rhythm/rhyme/alliteration produced, 1 indicated one rhythm/rhyme/alliteration produced, and 2 indicated two or more rhythms/rhymes/alliterations produced. The agreement index of independent judges was between 90 and 99%. In the data used in this study, Cronbach’s alpha coefficient for the composite measure of the phonological awareness context was 0.84.

#### 3.2.4. Second step of assessment in primary school—Writing skills

##### 3.2.4.1. Writing accuracy

A paper-and-pencil text dictation standardized for the Italian population (Tressoldi and Cornoldi, 2000) was used to measure writing accuracy in primary school children. The dictation was performed individually by children in a collective classroom session during school time. Based on the procedure in the manual, the children had to listen to a recorded text and to write down the text. We referred to the classification by Pinto et al. (2012) to identify the orthographic errors in text dictation. The classification allows one to identify the entire variability of orthographic errors that children may commit in Italian orthography, including the cases in which the pronunciation of the target word is preserved despite the spelling violation (e.g., “hanno” [have] instead of “anno” [year]), and the cases in which the pronunciation of the target word is changed due to a spelling violation (“tristezza” instead of “tristeza” [sadness]). The ratio between the total number of orthographic errors and the total number of written words produced the “writing accuracy” score (see, Pinto et al., 2012). According to the norms of this writing test, Cronbach’s alpha coefficient for the scale is 0.83.

#### 3.2.5. Second step of assessment in primary school—Reading skills

##### 3.2.5.1. Reading accuracy and reading speed

The MT reading test (Cornoldi et al., 1998) was used to measure reading accuracy and reading speed in primary-school children. It is a standardized test with strong psychometric properties administered by the experimenter to the children individually. Based on the procedure in the manual, the child is asked to read a text aloud as best as he/she could, while the experimenter registered the reading time and errors. The number of errors while reading aloud, such as mispronounced, or omitted, or added syllables produced the “reading accuracy” score. The ratio between the reading time in seconds and the total number of read syllables produced the “reading speed” score (e.g., the higher the score, the slower the children read). According to the norms of this reading test, Cronbach’s alpha coefficient for the scale is 0.70.

### 3.3. Data analysis

The main descriptive statistics for each variable (mean, standard deviation, minimum and maximum) and the Shapiro Wilk test for normality were calculated.

Preliminarily, Pearson’s bivariate correlations were performed to test the relationships between the home literacy variable and the emergent literacy outcome variables measured in the last year of preschool, i.e., phonological awareness and notational awareness, and the school performance outcome variables measured in first grade, i.e., reading accuracy, reading speed, and writing accuracy.

In view of the observed correlations, causal relationships were tested with multiple linear regressions. The checking of causal relationships of variables is a necessary condition to carry out mediational models. Indeed, first, the (simple) mediational model requires that the process by which a variable X (independent variable - IV) has an effect on Y (dependent variable - DV) can be described as follows: X has an effect on M, M has an effect on Y, and therefore X has an effect on Y because of the intervention of M. The mediational model holds if the mediating variable possesses certain characteristics such as M must be able to be caused by X (Hayes, 2013). Second, in the case of a parallel (multiple) mediation model, a distinguishing feature is the assumption that no mediator causally influences another. These mediators are allowed to correlate with one another, but not to influence each other in causality. With parallel mediation, we can test each proposed mediator taking into account the shared variance among them. However, overly correlated mediators can create multicollinearity, which affects the estimation of their partial relationships with the outcome variable (Hayes, 2013).

Considering that the mediating variables (phonological and notational awareness) have a reciprocal causal effect and that phonological awareness is not caused by home literacy (see regressions in Results), we decided to perform simple mediation analyses.

Therefore, three simple mediating models were run, in which the independent variable (IV) was home literacy, the dependent variables (DV) were outcomes in school performance, and the mediating variables (MV) were emergent literacy skills: (1) DV: reading accuracy, IV: home literacy and MV: notational awareness; (2) DV: reading speed, IV: home literacy and MV: notational awareness; and (3) DV: writing errors, IV: home literacy and MV: notational awareness.

For the indirect effects, the percentile bootstrap was used to derive robust estimates of standard errors and confidence intervals for regression coefficient estimates.

## 4. Results

Table 1 shows the main descriptive statistics for each variable (mean, standard deviation, minimum and maximum) and the Shapiro Wilk test for normality. The results are in line with previous studies having a similar population, in terms of age and level of schooling and socioeconomic background (e.g., Pinto et al., 2015; Incognito and Pinto, 2021).

**Table 1:** Descriptive statistics and Shapiro–Wilk coefficients.

	Mean (SD)	Minimum	Maximum	Shapiro–Wilk test
Home literacy	1.87 (0.78)	0	3	0.93***
Phonological awareness	1.62 (0.49)	0	2	0.75***
Notational awareness	2.38 (0.71)	0	3	0.82***
Reading accuracy	1.66 (2.24)	0	12	0.73***
Reading speed	1.31 (0.46)	0.39	2.57	0.96*
Writing accuracy	3.58 (4.30)	0	27	0.62***

*p < 0.05; ***p < 0.001.

Table 2 shows the results of bivariate correlations with Pearson’s coefficient. The results show that home literacy is significantly correlated with both emergent literacy skills, i.e., phonological awareness (*r* = 0.58, *p* < 0.01), notational awareness (*r* = 0.55, *p* < 0.01), and formalized literacy skills, i.e., reading speed (*r* = −0.32, *p* < 0.05), writing accuracy (*r* = 0.23, *p* < 0.05), except for reading accuracy (*r* = 0.02, *p* = n.s.). All significant correlations are positive, except for reading speed in which correlation is negative. Therefore, the higher the level of home literacy, the higher the performance in emergent and formalized literacy tasks.

**Table 2:** Pearson’s correlational bivariate analysis.

	Home literacy	Phonological awareness	Notational awareness	Reading accuracy	Reading speed	Writing accuracy
Home literacy	-	0.58**	0.55**	−0.02	−0.32*	0.23*
Phonological awareness		-	0.53**	−0.05	−0.30*	0.03
Notational awareness			-	−0.11	−0.30*	0.29*
Reading accuracy				-	0.30*	−0.13
Reading speed					-	0.32*
Writing accuracy						-

**Correlation is significant at the 0.01 level (two-tailed). *Correlation is significant at the 0.05 level (two-tailed).

Multiple linear regressions were run to test the model’s assumptions of mediation (simple or parallel) analysis. The results of the regressions show that phonological and notational awareness affect each other and that home literacy has no effect on phonological awareness (Table 3).

**Table 3:** Multiple linear regression analyses.

		*β* ^1^	*t*	*p*-Value	*R*-squared
DV: Phonological awareness	Home literacy	0.15	1.52	0.13	0.30***
	Notational awareness	0.46	4.78	<0.001	
DV: Notational awareness	Home literacy	0.38	4.57	<0.001	0.41***
	Phonological awareness	0.39	4.78	<0.001	

^1^Standardized coefficients; ***p < 0.001.

The simple mediating models were run separately for each formalized literacy variable.

**Figure 1:**
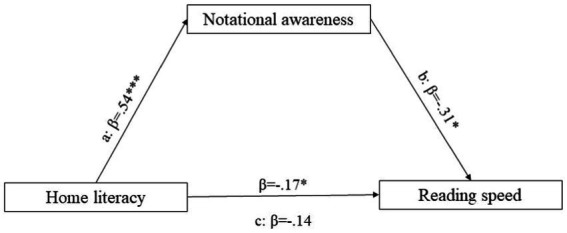
Mediation model: Home literacy (IV), notational awareness (MV) and reading speed (DV). ***Means that the effect is significant at *p* < 0.001; ** the effect is significant at *p* < 0.01; * the effect is significant at *p* < 0.05. All presented effects are unstandardized; a is the effect of home literacy on notational awareness; b is the effect of notational awareness on reading speed; β is the indirect effect of home literacy on reading speed; c is the total effect of home literacy on reading speed.

**Figure 2:**
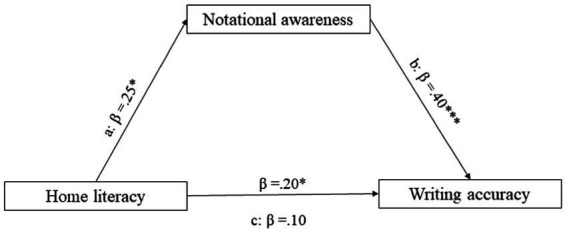
Mediation model: Home literacy (IV), notational awareness (MV) and writing accuracy (DV). ***Means that the effect is significant at *p* < 0.001; **the effect is significant at *p* < 0.01; *the effect is significant at *p* < 0.05. All presented effects are unstandardized; a is the effect of home literacy on notational awareness; b is the effect of notational awareness on writing accuracy; β is the indirect effect of home literacy on writing accuracy; c is the total effect of home literacy on writing accuracy.

Notational awareness turns out to be total mediator in the relationship between home literacy and reading speed (Figure 1) and writing accuracy (Figure 2). In both cases, the indirect effect is significant at *p* < 0.05, but total effect is not. Mediation occurs if the effect of the independent variable on the dependent variable is reduced (partial mediation) or canceled (total mediation) when the mediator is included (Hayes, 2009).

In line with Preacher and Hayes (2008), bootstrapping results showed that the indirect effect of home literacy on reading speed *via* notational awareness was significant (Lower C.I: −0.21; Upper C.I: −0.01), since zero was not included in the 95% confidence interval.

In the same way, bootstrapping results showed that the indirect effect of home literacy on writing accuracy *via* notational awareness was significant (Lower C.I: 0.10; Upper C.I: 1.10), since zero was not included in the 95% confidence interval.

## 5. Discussion

This longitudinal study provides results on the developmental relationships between home literacy and emergent literacy skills assessed in preschoolers, and their later reading and writing skills, assessed 1 year later when the same children attended primary school. Following Bronfenbrenner (1979) ecological theory and the field of research on emergent literacy (Sénéchal and LeFevre, 2002), theoretically, the study extended previous research on the connections between home literacy, emergent literacy skills, reading and writing outcomes by bridging significant life contexts such as home, preschool, and formalized schooling in a unitary way. Empirically, the study’s findings added to the limited literature of precursors of reading and writing development by testing the mediating role of emergent literacy skills in the relationship between early home literacy and later reading and writing skills in a transparent orthography like Italian. The essential role of the emergent literacy period for the development of early reading and writing in primary school in children learning Italian which has a transparent orthography emerged in the literature (Pinto et al., 2017) was confirmed by our results. A large part of previous studies (e.g., Kim, 2009) mainly focused on investigating direct relationships between home literacy and later reading and writing skills. Few studies considered indirect and mediational pathways (e.g., Inoue et al., 2018). Our results provide significant developmental and mediational relationships between home literacy, emergent literacy skills, and later reading and writing skills in a transparent orthography.

Prior to considering the results of the mediational models, the results of correlation analyses provide interesting insights about the connections *within* the period of emergent literacy and *between* emergent and formalized literacy periods.

Regarding the connections *within* the period of emergent literacy, the results of correlation analyses showed preschoolers who have higher scores in their HLE&P have higher notational awareness denoting higher availability in their memory of the orthographic representation of the letters of a word and they are able to write letter-like sign on a sheet of paper. Also, preschoolers who have higher scores in their HLE&P have higher phonological awareness denoting higher ability to detect sound units in language flow and to intentionally handle them. The richness of home environment, in terms of language and literacy stimuli and materials provided by the family, and the quantity and quality of child-parents’ language and literacy practices (Niklas and Schneider, 2015) is associated with higher notational awareness and phonological awareness, which are emergent literacy skills essential for developing adequate reading and writing skills at school (Pinto et al., 2012). This result is in line with studies conducted in a transparent language system such as Italian (Incognito and Pinto, 2021) and other language systems (Kim, 2009) that showed links between home literacy and emergent literacy skills.

Regarding the connections *between* emergent and formalized literacy periods, the results of correlation analyses showed preschoolers who have higher scores in their HLE&P have higher reading and writing skills, measured 1 year later in primary school. Although studies have demonstrated significant linkages between home literacy environment and reading in different orthographies, such as English (e.g., Sénéchal and LeFevre, 2014), our results contribute to expanding our understanding of these linkages in a transparent orthography like Italian.

The most innovative contributions are the results of the mediational analyses that showed the key role of preschoolers’ notational awareness in mediating the relationship between home literacy and formalized literacy skills. This salience of notational awareness is not surprising and supports previous research conducted in transparent orthographies (Pinto et al., 2012; Bigozzi et al., 2016b) which showed that notational awareness is fundamental for writing and reading tasks, and its deficiency is a predictor of dyslexia. Notational awareness in preschool children is a crucial ability because it allows a coding of sound to written signs and to connect them to each other. To develop notational awareness, it is necessary to develop a sensitivity of sound-sign integration which may be supported by home and preschool (Incognito et al., 2021). Children’s HLE&P can vary (Sénéchal and LeFevre, 2002; Manolitsis et al., 2011). From an early age, at home children might participate in home literacy practices and activities important for literacy development such as joint parent–child reading, telling and inventing stories, that implicitly provide a knowledge about sound-sign integration or the opportunity of reflecting on language. Beyond home, in preschool children develop and improve their notational awareness. It emerged as a concatenated effect between home literacy, preschool, and primary school. Indeed, our findings suggest that, within the preschooler group, children who grow up in families with higher levels of home literacy environment and whose parents tend to engage in discourse about the books written system or literacy aspects tend to have higher notational skills in preschool which support their reading and writing skills in primary school. This type of mediation of preschoolers’ notational skills might facilitate their later literacy development, especially in Italian in which there is a biunivocal correspondence between sound-sign.

A particularly innovative finding was that only notational awareness acts as a significant mediator in the relationship between preschoolers’ home literacy and their later reading and writing skills in primary school, and not phonological awareness. In accordance with Bigozzi’s earlier findings (2016), this phenomenon can be explained in that phonological awareness involves only the verbal channel (rhymes, oral alliteration) whereas notational awareness specifically refers to the relationship between sound and sign. Although phonological awareness is an important emergent literacy skill, its contribution to literacy development is particularly evident in opaque orthographies that require a strong sensitivity to different sounds forming words. Our results indicate that preschoolers’ phonological awareness is associated with later reading and writing skills, but that it did not act as mediator in the relationship between home literacy and subsequent literacy acquisitions. Another important line of reasoning might refer to the fact that in the Italian alphabetic language the correspondence is phoneme-grapheme, whereas the meta-phonological activity done at home is more at the level of syllables and phoneme and grapheme groups/patterns, i.e., the home literacy practices referring to phonological competence work at a less analytical level than that required for reading and writing, e.g., the parent plays the rhyming game rather than asking the child what happens if he removes a phoneme from a word. Schooling acts systematically through targeted work on phonology rather than what happens at home.

These findings advance our understanding of the important role of early HLE&P and notational skills for children’s reading and writing skills in a transparent orthography like Italian. Two major practical implications can be drawn from our findings. First, the importance of the level of home literacy for the development of emergent literacy skills in preschool and for the development of reading and writing in primary school should be recognized. Appropriate actions, such as promoting awareness in parents about the importance of home literacy should be nurtured in Italy, as well as in other countries, to ensure that children have the opportunity at home to engage with language and literacy activities with parents. Second, future studies should put their emphasis on how best to design preschool intervention programs so as to maximize preschoolers’ emergent literacy skills (Incognito et al., 2021), especially notational awareness which had a significant mediational role in our findings, in connection with families. The instruments used to assess HLE&P allow us to obtain useful information in view of the preparation of appropriate interventions to promote literacy development.

### 5.1. Limitations and future research

From a methodological point of view, the collection of information about HLE&P was based on a self-report questionnaire. We are aware that data based on multiple observations or interviews could provide stronger evidence of home literacy levels. In this respect, also to add the use of written parental reports on how and the extent to which they engage their children in literacy activities could give us a better idea about home literacy practices. Future studies should also consider the influence of both contextual and cognitive predictors of literacy development in preschool children simultaneously (see, e.g., Bonifacci et al., 2022). Furthermore, a longitudinal study with follow up which could identify children with reading or writing difficulties at the end of the second grade of primary school should give us a fuller picture about the relationships between home literacy, emergent literacy skills, and later achievements in reading and writing at school.

## Data availability statement

The raw data supporting the conclusions of this article will be made available by the authors, without undue reservation.

## Ethics statement

The studies involving human participants were reviewed and approved by the University of Florence Ethics Committee. Written informed consent to participate in this study was provided by the participants’ legal guardian/next of kin.

## Author contributions

All authors listed have made a substantial, direct, and intellectual contribution to the work and approved it for publication.

## Conflict of interest

The authors declare that the research was conducted in the absence of any commercial or financial relationships that could be construed as a potential conflict of interest.

## Publisher’s note

All claims expressed in this article are solely those of the authors and do not necessarily represent those of their affiliated organizations, or those of the publisher, the editors and the reviewers. Any product that may be evaluated in this article, or claim that may be made by its manufacturer, is not guaranteed or endorsed by the publisher.

## Footnotes

^1^ https://www.istat.it/it/files//2022/12/Istat-Audizione-Bilancio-2023.pdf

## References

[ref2] Albuquerque A. Alves Martins M. (2022). Invented spelling as a tool to develop early literacy: the predictive effect on reading and spelling acquisition in Portuguese. J. Writ. Res. 14, 109–127. doi: 10.17239/jowr-2022.14.01.04

[ref3] Bigozzi L. Tarchi C. Pezzica S. Pinto G. (2016a). Evaluating the predictive impact of an emergent literacy model on dyslexia in Italian children: a four-year prospective cohort study. J. Learn. Disabil. 49, 51–64. doi: 10.1177/0022219414522708, PMID: 24608754

[ref4] Bigozzi L. Tarchi C. Pinto G. Accorti Gamannossi B. (2016b). Predicting dyslexia in a transparent orthography from grade 1 literacy skills: a prospective cohort study. Read. Writ. Q. 32, 353–372. doi: 10.1080/10573569.2014.988310

[ref5] Bonifacci P. Trambagioli N. Bernabini L. Tobia V. (2022). Home activities and cognitive skills in relation to early literacy and numeracy: testing a multifactorial model in preschoolers. Eur. J. Psychol. Educ. 37, 681–705. doi: 10.1007/s10212-021-00528-2

[ref6] Bronfenbrenner U. (1979). Contexts of child rearing: problems and prospects. Am. Psychol. 34, 844–850. doi: 10.1037/0003-066X.34.10.844

[ref800] Cornoldi C. Colpo G. MT Group (1998). Prove di lettura MT per la scuola elementare-2 [MT reading tests for primary school]. Florence, Italy: Organizzazioni Speciali.

[ref7] Dowker A. Pinto G. (1993). Phonological devices in poems by English and Italian children. J. Child Lang. 20, 697–706. doi: 10.1017/S0305000900008540, PMID: 8300782

[ref8] Fritjers J. C. Barron R. W. Brunello M. (2000). Direct and mediated influences of home literacy and literacy interest on pre-readers’ oral vocabulary and early written language skill. J. Educ. Psychol. 92, 466–477. doi: 10.1037/0022-0663.92.3.466

[ref9] Georgiou G. K. Torppa M. Manolitsis G. Lyytinen H. Parrila R. (2012). Longitudinal predictors of reading and spelling across languages varying in orthographic consistency. Read. Writ. 25, 321–346. doi: 10.1007/s11145-010-9271-x

[ref10] Hayes A. F. (2009). Beyond baron and Kenny: statistical mediation analysis in the new millennium. Commun. Monogr. 76, 408–420. doi: 10.1080/03637750903310360

[ref11] Hayes A. F. (2013). Introduction to Mediation, Moderation, and Conditional Process Analysis. A Regression-Based Approach. New York, NY: The Guilford Press.

[ref12] Incognito O. Bigozzi L. Vettori G. Pinto G. (2021). Efficacy of two school-based interventions on notational ability of bilingual preschoolers: a group-randomized trial study. Front. Psychol. 12:686285. doi: 10.3389/fpsyg.2021.686285, PMID: 34721139 PMC8553984

[ref13] Incognito O. Pinto G. (2021). Longitudinal effects of family and school context on the development of emergent literacy skills in preschoolers. Curr. Psychol., 1–11. doi: 10.1007/s12144-021-02274-6

[ref14] Inoue T. Georgiou G. K. Parrila R. Kirby J. R. (2018). Examining an extended home literacy model: the mediating roles of emergent literacy skills and reading fluency. Sci. Stud. Read. 22, 273–288. doi: 10.1080/10888438.2018.1435663

[ref15] Inoue T. Manolitsis G. de Jong P. F. Landerl K. Parrila R. Georgiou G. K. (2020). Home literacy environment and early literacy development across languages varying in orthographic consistency. Front. Psychol. 11:1923. doi: 10.3389/fpsyg.2020.01923, PMID: 32849130 PMC7412602

[ref16] Kim Y. S. (2009). The relationship between home literacy practices and developmental trajectories of emergent literacy and conventional literacy skills for Korean children. Read. Writ. 22, 57–84. doi: 10.1007/s11145-007-9103-9

[ref17] Kim S. Im H. Kwon K. A. (2015). The role of home literacy environment in toddlerhood in development of vocabulary and decoding skills in Child and Youth Care Forum, 44, 835–852. doi: 10.1007/s10566-015-9309-y

[ref18] Landerl K. Freudenthaler H. H. Heene M. De Jong P. F. Desrochers A. Manolitsis G.. (2019). Phonological awareness and rapid automatized naming as longitudinal predictors of reading in five alphabetic orthographies with varying degrees of consistency. Sci. Stud. Read. 23, 220–234. doi: 10.1080/10888438.2018.1510936

[ref19] Manolitsis G. Georgiou G. K. Parrila R. (2011). Revisiting the home literacy model of reading development in an orthographically consistent language. Learn. Instr. 21, 496–505. doi: 10.1016/j.learninstruc.2010.06.005

[ref20] Mol S. E. Bus A. G. (2011). To read or not to read: a meta-analysis of print exposure from infancy to early adulthood. Psychol. Bull. 137, 267–296. doi: 10.1037/a0021890 21219054

[ref21] Moll K. Ramus F. Bartling J. Bruder J. Kunze S. Neuhoff N.. (2014). Cognitive mechanisms underlying reading and spelling development in five European orthographies. Learn. Instr. 29, 65–77. doi: 10.1016/j.learninstruc.2013.09.003

[ref22] Niklas F. Schneider W. (2015). With a little help: improving kindergarten children’s vocabulary by enhancing the home literacy environment. Read. Writ. 28, 491–508. doi: 10.1007/s11145-014-9534-z

[ref600] Noble C. Sala G. Peter M. Lingwood J. Rowland C. Gobet F.. (2019). The impact of shared book reading on children’s language skills: A meta-analysis. Educational Research Review, 2:100290.

[ref23] Ouellette G. Sénéchal M. (2017). Invented spelling in kindergarten as a predictor of Reading and spelling in grade 1: a new pathway to literacy, or just the same road, less known? Dev. Psychol. 53, 77–88. doi: 10.1037/dev0000179 27617354

[ref24] Pinto G. Bigozzi L. Gamannossi B. A. Vezzani C. (2009). Emergent literacy and learning to write: a predictive model for Italian language. Eur. J. Psychol. Educ. 24, 61–78. doi: 10.1007/BF03173475

[ref25] Pinto G. Bigozzi L. Gamannossi B. A. Vezzani C. (2012). Emergent literacy and early writing skills. J. Genet. Psychol. 173, 330–354. doi: 10.1080/00221325.2011.609848, PMID: 22919895

[ref26] Pinto G. Bigozzi L. Tarchi C. Vezzani C. Accorti Gamannossi B. (2016). Predicting reading, spelling, and mathematical skills: a longitudinal study from kindergarten through first grade. Psychol. Rep. 118, 413–440. doi: 10.1177/0033294116633357, PMID: 27154371

[ref27] Pinto G. Bigozzi L. Vezzani C. Tarchi C. (2017). Emergent literacy and reading acquisition: a longitudinal study from kindergarten to primary school. Eur. J. Psychol. Educ. 32, 571–587. doi: 10.1007/s10212-016-0314-9

[ref28] Pinto G. Tarchi C. Bigozzi L. (2015). The relationship between oral and written narratives: a three-year longitudinal study of narrative cohesion, coherence, and structure. Br. J. Educ. Psychol. 85, 551–569. doi: 10.1111/bjep.12091, PMID: 26373247

[ref29] Preacher K. J. Hayes A. F. (2008). Assessing Mediation in Communication Research (pp. 13–54). London: The Sage Sourcebook of Advanced Data Analysis Methods for Communication Research.

[ref30] Rodriguez E. T. Tamis-LeMonda C. (2011). Trajectories of the home learning environment across the first 5 years: associations with children’s vocabulary and literacy skills at prekindergarten. Child Dev. 82, 1058–1075. doi: 10.1111/j.1467-8624.2011.01614.x, PMID: 21679179

[ref31] Sénéchal M. LeFevre J. A. (2002). Parental involvement in the development of children’s reading skill: a five-year longitudinal study. Child Dev. 73, 445–460. doi: 10.1111/1467-8624.00417, PMID: 11949902

[ref32] Sénéchal M. LeFevre J. A. (2014). Continuity and change in the home literacy environment as predictors of growth in vocabulary and reading. Child Dev. 85, 1552–1568. doi: 10.1111/cdev.12222, PMID: 24467656

[ref33] Sénéchal M. LeFevre J. A. Hudson E. Lawson E. P. (1996). Knowledge of storybooks as a predictor of young children’s vocabulary. J. Educ. Psychol. 88, 520–536. doi: 10.1037/0022-0663.88.3.520

[ref34] Sénéchal M. LeFevre J. A. Smith-Chant B. L. Colton K. V. (2001). On refining theoretical models of emergent literacy the role of empirical evidence. J. Sch. Psychol. 39, 439–460. doi: 10.1016/S0022-4405(01)00081-4

[ref35] Sénéchal M. Lefevre J. A. Thomas E. M. Daley K. E. (1998). Differential effects of home literacy experiences on the development of oral and written language. Read. Res. Q. 33, 96–116. doi: 10.1598/RRQ.33.1.5

[ref36] Silinskas G. Sénéchal M. Torppa M. Lerkkanen M. K. (2020). Home literacy activities and children’s reading skills, independent reading, and interest in literacy activities from kindergarten to grade 2. Front. Psychol. 11:1508. doi: 10.3389/fpsyg.2020.01508, PMID: 32733336 PMC7362993

[ref37] Tressoldi P. E. Cornoldi C. (2000). Batteria per la Valutazione Della Scrittura e Della Competenza Ortografica Nella Scuola Dell’obbligo (BVSCO, Battery for the Assessment of Writing Skills of Children from 7 to 13 Years Old). Florence: Giunti OS.

[ref38] Zhang S. Z. Inoue T. Shu H. Georgiou G. K. (2020). How does home literacy environment influence reading comprehension in Chinese? Evidence from a 3-year longitudinal study. Read. Writ. 33, 1745–1767. doi: 10.1007/s11145-019-09991-2

[ref39] Zhang X. Lau C. Su Y. (2021). Home environment and development of English as a second/foreign language for young children in Asian contexts: a systematic review and meta-analysis. Early Educ. Dev., 1–19. doi: 10.1080/10409289.2021.202006635082478

